# Effects of Chestnut Tannin Extract on Enteric Methane Emissions, Blood Metabolites and Lactation Performance in Mid-Lactation Cows

**DOI:** 10.3390/ani15152238

**Published:** 2025-07-30

**Authors:** Radiša Prodanović, Dušan Bošnjaković, Ana Djordjevic, Predrag Simeunović, Sveta Arsić, Aleksandra Mitrović, Ljubomir Jovanović, Ivan Vujanac, Danijela Kirovski, Sreten Nedić

**Affiliations:** 1 Department of Ruminant and Swine Diseases, Faculty of Veterinary Medicine, University of Belgrade, Bulevar Oslobodjenja 18, 11000 Belgrade, Serbia; prodanovic@vet.bg.ac.rs (R.P.); sarsic@vet.bg.ac.rs (S.A.); aleksandra.mitrovic@vet.bg.ac.rs (A.M.); vujanac@vet.bg.ac.rs (I.V.); sreten.nedic@vet.bg.ac.rs (S.N.); 2 Department of Physiology and Biochemistry, Faculty of Veterinary Medicine, University of Belgrade, Bulevar Oslobodjenja 18, 11000 Belgrade, Serbia; ljubomir.jovanovic@vet.bg.ac.rs (L.J.); dani@vet.bg.ac.rs (D.K.); 3 Department of Biochemistry, Institute for Biological Research “Siniša Stankovic”-National Institute of the Republic of Serbia, University of Belgrade, Bulevar Despota Stefana 142, 11000 Belgrade, Serbia; djordjevica@ibiss.bg.ac.rs; 4 Sabac Veterinary Station, Vojvode Putnika 52, 15000 Sabac, Serbia; simeunovic.p@gmail.com

**Keywords:** supplements, methane mitigation, hormonal status, metabolic status, milk yield, sustainability

## Abstract

Dietary tannin supplementation represents a potential strategy to modulate rumen fermentation and enhance lactation performance in dairy cows, though responses remain inconsistent. A 21-day feeding trial was conducted to evaluate the effect of chestnut tannin (CNT) extract on the enteric methane emissions (EME), blood metabolites, and milk production traits in mid-lactation dairy cows. Thirty-six Holstein cows were allocated to three homogeneous treatment groups: control (CNT_0_, 0 g/d CNT), CNT_40_ (40 g/d CNT), and CNT_80_ (80 g/d CNT). Measurements of EME, dry matter intake (DMI), milk yield (MY), and blood and milk parameters were carried out pre- and post-21-day supplementation period. Compared with the no-additive group, the CNT extract reduced methane production, methane yield, and methane intensity in CNT_40_ and CNT_80_ (*p* < 0.001). CNT_40_ and CNT_80_ cows exhibited lower blood urea nitrogen (*p* = 0.019 and *p* = 0.002) and elevated serum insulin (*p* = 0.003 and *p* < 0.001) and growth hormone concentrations (*p* = 0.046 and *p* = 0.034), coinciding with reduced aspartate aminotransferase (*p* = 0.016 and *p* = 0.045), and lactate dehydrogenase (*p =* 0.011 and *p =* 0.008) activities compared to control. However, CNT_80_ had higher circulating NEFA and BHBA than CNT_0_ (*p* = 0.003 and *p* = 0.004) and CNT_40_ (*p* = 0.035 and *p* = 0.019). The blood glucose, albumin, and total bilirubin concentrations were not affected. MY and fat- and protein-corrected milk (FPCM), MY/DMI, and FPCM/DMI were higher in both CNT_40_ (*p* = 0.004, *p* = 0.003, *p* = 0.014, *p* = 0.010) and CNT_80_ (*p* = 0.002, *p* = 0.003, *p* = 0.008, *p* = 0.013) cows compared with controls. Feeding CNT_80_ resulted in higher protein content (*p* = 0.015) but lower fat percentage in milk (*p* = 0.004) compared to CNT_0_. Milk urea nitrogen and somatic cell counts were significantly lower in both CNT_40_ (*p* < 0.001, *p* = 0.009) and CNT_80_ (*p* < 0.001 for both) compared to CNT_0_, while milk lactose did not differ between treatments. These findings demonstrate that chestnut tannin extract effectively mitigates EME while enhancing lactation performance in mid-lactation dairy cows.

## Simple Summary

The growing demand for sustainable livestock production highlights the urgent need to reduce enteric methane (CH_4_) emissions, a major contributor to agricultural greenhouse gases. At the same time, dairy producers are challenged to maintain animal health while improving productivity. Among nutritional solutions, plant-derived compounds like tannins have gained interest for their potential to modulate rumen fermentation and protein metabolism. In our study, supplementing dairy cows with chestnut tannin extract led to reduced CH_4_ production and improvement in lactation response. These results suggest a promising step toward more climate-friendly and efficient dairy farming.

## 1. Introduction

The agriculture sector contributes considerably to global greenhouse gas (GHG) emissions. As the largest source of agricultural GHG emissions, enteric CH_4_ accounts for 1.6 to 2.7 GtCO_2_e annually, with a global warming potential 28 times greater than that of carbon dioxide (CO_2_) [1,2]. Beyond its environmental impact, CH_4_ emissions also represent production efficiency and economic concerns due to the loss of ingested metabolic energy, which ranges from 3.9% to 10.7% [3,4].

Considering this problem, numerous international organizations and research centers are searching for nutritional strategies that will cover important aspects of ruminant production to reduce the enteric CH_4_ emissions. Dairy cows, as major contributors to CH_4_ emissions in livestock farming, serve as a critical model for evaluating such strategies due to their complex metabolic pathways, which are influenced by intrinsic and extrinsic factors [5]. Nutritional strategies to reduce enteric CH_4_ emissions should not compromise animal health and productivity [5]. An ideal nutritional intervention should simultaneously reduce CH_4_ emissions and enhance production efficiency [6]. In this context, tannins—particularly hydrolysable tannins (HT)—have emerged as a promising candidate.

Tannins are water-soluble polyphenolic compounds capable of binding proteins, amino acids, and polysaccharides [7]. Classified into condensed tannins (CT), hydrolysable tannins (HT), and phlorotannins (PT), HT—especially those derived from sweet chestnut (*Castanea sativa* Mill.)—have demonstrated potential in reducing ruminal CH_4_ production [8,9,10]. Proposed mechanisms include (1) decreased organic matter digestibility, particularly fiber [11,12,13], and (2) direct inhibition of methanogenic *Archaea* and *Protozoa* [14,15]. While reduced fiber degradation may lower acetate-derived CH_4_ [16], studies indicate that HT exhibits stronger CH_4_ mitigation potential than CT, with less adverse effects on fiber digestibility [9]. Notably, chestnut tannin (CNT) extract has been shown to suppress methanogenesis without impairing acetate production [17,18] likely due to selective inhibition of *Methanobrevibacter* spp. [19].

Thanks to its hydrophobic and ionic interactions (predominantly via hydrogen bonds), CNT can form complexes with proteins from feed, rumen bacteria, and extracellular enzymes [20]. This results in reduced protein degradability, ammonia production, and volatile fatty acids concentrations in the rumen [14,21,22], resulting in increased protein and amino acid flow to the lower gut [9]. CNT also exhibits antioxidant, anti-inflammatory, immunomodulatory, antimicrobial, and toxin-binding properties, with well-documented positive effects on gut and overall health and reproduction [23,24,25]. However, while CT have been extensively studied in ruminant nutrition [26,27,28], there are limited number of studies that dealt with the impact of CNT on production parameters in dairy cows, showing either no significant effects of supplementation on dry matter intake, milk yield, and milk quality [18,29,30], or an improvement of milk production and quality [31,32].

This study builds upon prior research demonstrating that dietary CNT supplementation during the close-up dry period enhances metabolic adaptation and colostrum quality in dairy cows [23]. In particular, the positive effect of dietary chestnut tannins on the immunometabolic status of dairy cows was mediated through altered hormonal status near parturition [33].

We hypothesized that supplementation with CNT reduces enteric CH_4_ emissions and improves milk production traits during mid-lactation, which could be mediated through altered lactogenic hormones (such as insulin and growth hormone), promoting more efficient nutrient partitioning toward milk synthesis. Therefore, the present study aimed to evaluate the efficacy of CNT in reducing CH_4_ emissions and in improving the lactation performance using mid-lactating Holstein dairy cows.

## 2. Materials and Methods

### 2.1. Experimental Site and Ethics Statement

The study was carried out on a commercial Holstein farm (PIK Bečej) in the northern part of Serbia, City of Bečej, at latitude 45.593790 North, longitude 19.978214 East. The experimental protocol received formal approval from the Veterinary Directorate of the Ministry of Agriculture, Forestry and Water Management of the Republic of Serbia (approval no. 323-07-11720/2020-05/4) under national animal welfare regulations.

### 2.2. Animals, Dietary Treatments, and Study Design

Thirty-six clinically healthy and mid-lactation Holstein cows were chosen and divided into three numerically equal (*n* = 12) diet groups: CNT_0_ (cows fed a basal diet with no additional supplementation), CNT_40_ (cows supplemented with 40 g/day of chestnut tannin extract), and CNT_80_ (cows supplemented with 80 g/day of chestnut tannin extract). The groups of cows were balanced for body weight (CNT_0_: 618.8 ± 19.1 kg; CNT_40_: 615.0 ± 26.4 kg; CNT_80_: 620.2 ± 24.0 kg), days in milk (CNT_0_: 114.3 ± 4.9; CNT_40_: 113.3 ± 4.6; CNT_80_: 112.7 ± 3.8), milk yield (CNT_0_: 27.4 ± 3.3 kg/d; CNT_40_: 28.1 ± 2.7 kg/d; CNT_80_: 28.0 ± 3.1 kg/d), dry matter intake (CNT_0_: 21.7 ± 0.1 kg/d; CNT_40_: 21.5 ± 0.1 kg/d; CNT_80_: 21.6 ± 0.2 kg/d), and enteric CH_4_ production (CNT_0_: 94.3 ± 3.6 ppm; CNT_40_: 93.3 ± 3.2 ppm; CNT_80_: 92.7 ± 3.5 ppm) at the beginning of the study. Dietary treatment lasted for 21 consecutive days, and the daily dose of chestnut tannins was divided in half and delivered to cows with morning and evening total mixed rations (TMR), as described by Prodanović et al. [34]. The chestnut tannin extract used in this study is commercially available (Tanimil SCC, Tanin Sevnica, Sevnica, Slovenia) and contains 48% hydrolisable and 2.1% condensed tannins embedded in fiber left from tannin extraction.

**Figure 1:**
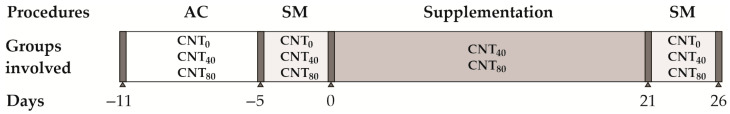
Scheme of the experimental design. CNT_0_—no supplemented cows; CNT_40_—cows supplemented with 40 g of chestnut tannin extract; CNT_80_—cows supplemented with 80 g of chestnut tannin extract; AC—acclimation period; SM—sampling and measurement period.

The study design employed a sampling and measurement scheme adapted from those of Van Wesemael et al. [35] and Bošnjaković et al. [36]. Before the start of the study, all cows underwent a 7-day acclimation (AC) period. In addition, two sampling and measurement (SM) periods, each lasting 5 days, were conducted: one immediately before and one immediately after the 21-day supplementation with chestnut tannins. During each SM period, the procedures were performed in the following order and duration: dry matter intake (DMI) and milk yield (MY) were recorded every day to obtain a reliable average per period, enteric CH_4_ emissions were measured for four consecutive days to receive a reliable estimation per period, and venous blood and milk samples were collected once per period (Figure 1).

The cows were kept in the same husbandry conditions (tie stall barn). All cows were fed the same basal diet (Table 1), which met or exceeded the National Research Council Requirements for Dairy Cattle [37] and administered in equal portions as TMR twice daily at 6:30 am and 5:30 pm. Water consumption was ad libitum via automatic water bowls. The health of the cows was clinically examined daily by the farm veterinarian and study researchers. These included evaluations of behavior, general appearance, and visible health indicators. No signs of any disease or tannin toxicity were detected before, during, or after the study. In addition, there was no negative impact of dietary treatments on DMI at the end of the trial (CNT_0_: 21.5 ± 0.1 kg/d; CNT_40_: 21.7 ± 0.1 kg/d; CNT_80_: 21.8 ± 0.3 kg/d).

### 2.3. Measuring Enteric CH_4_ Emissions

The measurement of the enteric CH_4_ emissions was performed following the protocols described by Niero et al. [38] and Bošnjaković et al. [36]. In brief, measurement of the enteric CH_4_ emissions was performed twice daily (2–4 and 6–8 h after morning feeding) for 4 consecutive days in the nostril area of the cows using a hand-held laser methane detector (LMD Mini-Green; Tokyo Gas Engineering Solutions, Tokyo, Japan) [39]. A single measurement continuously lasted 4 min, and LMD recorded CH_4_ concentrations at 0.5 s intervals, providing the CH_4_ concentration profile of 480 values for each cow per measurement. The LMD measures CH_4_ concentration in parts per milion meter (ppm × m). However, a 1 m distance between LMD and cow’s nostrils allowed a direct conversion to ppm [40]. Before each enteric CH_4_ measurement session, the LMD was connected to the operator’s mobile phone via Bluetooth. Thus, actual CH_4_ concentrations were stored in the Android (Google Play) mobile application LeakFinder (v.1.29 (49)), enabling further data processing. LMD data processing involved the inspection of each CH_4_ profile. The lowest CH_4_ value in the profile was considered a background concentration and was subtracted from all other individual values of the respective data set, as described by Sorg [41]. The average of all CH_4_ values was chosen in this study to compare enteric CH_4_ emissions’ phenotypes among the examined groups of cows, as described by Grobler et al. [42] and Niero et al. [38]. Finally, the two parameters of CH_4_ emissions data were derived in this study, including CH_4_ production (CH_4_, ppm), CH_4_ yield (CH_4_/DMI, ppm/kg), and CH_4_ intensity per kg of FPCM (CH_4_/FPCM, ppm/kg), as proposed by Grešáková et al. [43].

### 2.4. Blood Samples and Analyses

Blood was collected before the morning feeding by puncturing the jugular vein into Vacutainer blood collection tubes (Becton Dickinson, Plymouth, UK) with a clot activator for serum separation. The tubes were immediately placed in an ice box and transferred to the laboratory within one hour. Blood samples were centrifuged at 1800× *g* for 10 min, and aliquoted into 2 mL microfuge tubes. Aliquots of serum were stored at −20 °C until analyzed for non-esterified fatty acids (NEFA), beta hydroxybutyrate (BHBA), blood urea nitrogen (BUN), albumin, aspartate aminotranspherase (AST), lactate dehydrogenase (LDH), total bilirubin, insulin and growth hormone. Biochemical metabolites were analyzed by the Department of Ruminants and Swine Diseases (Belgrade, Serbia) using the respective kits: NEFA (colorimetric method) and BHBA (enzymatic method), both from Randox Laboratories Ltd. (Crumlin, UK); albumin (Bromcresol green method), BUN (urease/glutamate dehydrogenase method), total bilirubin (diazotized sulphanilic acid method), AST (IFCC method), and LDH (pyruvate method) from BioSystems S.A. (Barcelona, Spain). Analyses were performed automatically by spectrophotometry (A15; BioSystems S.A., Barcelona, Spain). Glucose concentration was also measured in whole blood enzymatically (glucose dehydrogenase, GDH-NAD method) using commercial test strips (Abbott Diabetes Care Ltd., Oxon, UK). Insulin concentration was determined by radioimmunoassay technique using commercially available RIA kits (INEP-Zemun, Belgrade, Serbia) validated for use with bovine serum. The mean Intra-Assay coefficient of variation (CV) for duplicate sample was 4.7%, while Inter-Assay CV was <10%. Somatotropin concentrations were measured in serum samples by bovine specific ELISA kit (E2278Bo, Bioassay Technology Laboratory, Nanhu Dist., Jiaxing, Zhejiang, China) according to the manufacturer’s instructions. Absorbance at 450 nm was measured spectrophotometrically (Multiskan Spectrum, Thermo, Vantaa, Finland). Somatotropin concentrations were determined using regression analysis curve-fitting method (GraphPad Prism 10.0, Inc., San Diego, CA, USA) and given as ng/mL. Kit sensitivity was 0.017 ng/mL, while Intra-Assay coefficient of variation (CV) was <8%, and Inter-Assay CV was <10%.

### 2.5. Milk Samples and Analyses

Milk samples from morning and evening milking were collected, pooled, and stored at 4 °C until analyzed for fat, protein, lactose, milk urea nitrogen (MUN), and somatic cell counts (SCC). The milk composition and SCC were determined by infrared analysis using MilkoScan FT 600 and Fossomatic 500 Basic (Foss Electric, Integrated Milk Testing^TM^, Hillerød, Denmark), respectively. Yield of fat- and protein-corrected milk (FPCM) was also expressed and calculated as milk (kg/d) × [0.1226 × fat (%) + 0.0776 × true protein (%) + 0.2534] according to International Dairy Federation [44]. Feed conversion efficiencies were calculated by dividing milk and FPCM yields by DMI.

### 2.6. Data Analysis

The data were statistically analyzed using SPSS Statistics 29.0 software (IBM^®^, Armonk, NY, USA). The normality assumption was checked with the Shapiro–Wilk test following Ferlizza et al. [45] and only the milk SCC had to be logarithmically transformed (Log_10_ SCC). Following the protocol of Botaro et al. [46] SCC was further anti-log_10_ transformed to present the results. To statistically test the influence of the dietary treatment on the examined parameters, analysis of variance was applied with dietary treatment as an independent factor and the examined variable (post-supplementation) as dependent variable, while the pre-supplementation values were included in the model as covariates [36]:

*Y_ij_* = *µ* + *τ_i_* + *β*(*X_ij_* − *X*) + *ε_ij,_*

where *Y_ij_* is the dependent variable, *μ* is the overall mean, *τ_i_* is the fixed effect of the treatment, *X_ij_* is the covariate (baseline values before the supplementation), *X* is the overall mean of the covariate, *β* is the regression coefficient for the covariate, and *ε_ij_* is the random error term. Fisher’s Protected Least Significant Difference was used to compare treatment means as described by Griffiths et al. [47]. In addition, to test for linear and quadratic effects of tannin level in the diet, preplanned orthogonal polynomial contrasts were used, similar to Aguerre et al. [48] and Vargas-Ortiz et al. [49]:

$$C=\sum_{i=1}^{k}ci\cdot Yi,$$

where *C* is the contrast value, ci are the contrast coefficients, *Yi* is the mean of the dependent variable, and *k* is the number of treatment levels (in this case, *k* = 3). All data were expressed as mean ± standard error (SE). Significance was declared at *p* < 0.05.

## 3. Results

### 3.1. Indicators of Enteric CH_4_ Emissions

Table 2 shows significant differences in CH_4_ production, CH_4_ yield and CH_4_ intensity among treatments (*p* < 0.001, respectively). CNT_40_ and CNT_80_ cows had significantly lower CH_4_ production (*p* < 0.001 for both), CH_4_ yield (*p* < 0.001 for both), and CH_4_ intensity (*p* < 0.001 for both) than the CNT_0_ cows. Specifically, CH_4_ production, CH_4_ yield, and CH_4_ intensity were reduced by 23%, 24%, and 29% in CNT_40_ cows, and by 21%, 22%, and 26% in CNT_80_ cows, compared to CNT_0_ cows.

The effect of chestnut tannins supplementation on CH_4_ production, CH_4_ yield, and CH_4_ intensity showed both significant linear (*p* = 0.001, *p* < 0.001 and *p* < 0.001, respectively) and quadratic (*p* = 0.011, *p* = 0.008 and *p* = 0.005, respectively) responses.

### 3.2. Blood Biomarkers

#### 3.2.1. Metabolism

Concentrations of BUN (*p* = 0.006), NEFA (*p* = 0.010), BHBA (*p* = 0.009), and insulin (*p* < 0.001) were significantly affected by the treatment (Table 3). Thus, CNT_40_ (*p* = 0.019) and CNT_80_ (*p* = 0.002) cows had significantly lower BUN concentrations than CNT_0_ cows. Concentrations of NEFA and BHBA were significantly higher in CNT_80_ cows compared to both CNT_40_ (*p* = 0.035 for NEFA; *p* = 0.019 for BHBA) and CNT_0_ (*p* = 0.003 for NEFA; *p* = 0.004 for BHBA) cows. Although insulinemia was significantly lower in CNT_40_ than that of CNT_80_ cows (*p* = 0.001), CNT_40_ had significantly higher insulin levels compared to CNT_0_ cows (*p* = 0.003). In addition, both CNT_40_ (*p* = 0.046) and CNT_80_ (*p* = 0.034) cows had significantly higher levels of growth hormone compared to CNT_0_ cows.

Significant linear response to the chestnut tannins treatment was found for BUN (*p* = 0.005), NEFA (*p* = 0.002), BHBA (*p* = 0.004), insulin (*p* < 0.001) and growth hormone (*p* = 0.027) concentrations.

#### 3.2.2. Liver Function

Among the indicators of liver function examined in this trial, only AST (*p* = 0.037) and LDH (*p* = 0.011) were significantly affected by the treatment (Table 4). In this regard, CNT_0_ cows exhibited significantly higher activities of AST and LDH compared to CNT_40_ (*p* = 0.016 for AST; *p* = 0.011 for LDH) and CNT_80_ (*p* = 0.045 for AST; *p* = 0.008 for LDH) cows. These parameters also showed a significant linear response (*p* = 0.043 for AST and *p* = 0.007 for LDH) to the chestnut tannins supplementation.

### 3.3. Milk Production Traits

#### 3.3.1. Milk Production

There was a significant influence of dietary treatment on MY (*p* = 0.003), FPCM (*p* = 0.003), FE_MY_ (*p* = 0.014), and FE_FPCM_ (*p* = 0.015). Accordingly, both CNT_40_ and CNT_80_ showed significantly higher MY (*p* = 0.004 for CNT_40_; *p* = 0.002 for CNT_80_), FPCM (*p* = 0.003 for both), FE_MY_ (*p* = 0.014 for CNT_40_; *p* = 0.008 for CNT_80_), and FE_FPCM_ (*p* = 0.010 for CNT_40_; *p* = 0.013 for CNT_80_) than CNT_0_ cows (Table 4). Significant linear response to the chestnut tannins treatment was found for MY (*p* < 0.001), FPCM (*p* = 0.003), FE_MY_ (*p* = 0.003) and FE_FPCM_ (*p* = 0.013).

#### 3.3.2. Milk Composition

Contents of milk fat (*p* = 0.023), milk protein (*p* = 0.001), and concentrations of MUN (*p* < 0.001) were significantly affected by the dietary treatment (Table 4). Therefore, milk fat content was significantly lower (*p* = 0.004), while milk protein content was significantly higher (*p* = 0.015) in CNT_80_ than in CNT_0_ cows. In addition, CNT_0_ cows had significantly higher MUN concentrations compared with CNT_40_ (*p* < 0.001) and CNT_80_ (*p* < 0.001) cows. Only the milk fat (*p* < 0.001) and protein (*p* = 0.004) contents showed linear response to the chestnut tannins supplementation.

#### 3.3.3. Somatic Cell Count

Dietary treatment had a significant influence (*p* < 0.001) on SCC, with significanlty lower SCC in both CNT_40_ (*p* = 0.009; 350,800 SC/mL) and CNT_80_ (*p* < 0.001; 204,400 SC/mL) compared to CNT_0_ (487,500 SC/mL) cows, showing also a linear effect (*p* = 0.001) to the response of the treatments (Table 5).

## 4. Discussion

Reducing greenhouse gas (GHG) emissions, particularly CH_4_ and nitrogenous compounds, is essential for dairy producers to address societal concerns regarding the environmental impact of dairy farming while enhancing animal performance through optimized rumen fermentation pathways [6]. In this context, natural substances like tannins have demonstrated encouraging outcomes [23,34,50]. However, previous results have shown a large proportion of variation in the effectiveness of tannins in reducing enteric CH_4_ emissions in both in vitro and in vivo studies. Key factors influencing this variability include tannin type and dosage, basal diet composition, duration of dietary intervention, and farm management practices [10,47,50]. These elements can affect the ruminal metabolome and microbiome, thereby impacting the effectiveness of the treatment [51,52].

In the present study, spot measurements of CH_4_ in the exhaled air around the cattle’s mouth and nostrils were performed to determine whether chestnut tannin extract is effective when included in TMR. The obtained results aligned with our hypothesis and showed that both doses of CNT extract are effective in reducing the enteric CH_4_ emissions without compromising the health and performance of mid-lactation cows. For both CNT groups, a significant reduction in all three indicators of enteric CH_4_ emissions, including CH_4_ production, CH_4_ yield and CH_4_ intensity, was achieved at the end of the supplementation period. Namely, a decrease in CH_4_ production of 23% and 21% was recorded for CNT_40_ and CNT_80_, respectively, compared to CNT_0_. Similarly, reduction percentages of the CH_4_ yield and CH_4_ intensity were 24% and 29% in CNT_40_, respectively, and 22% and 26% in CNT_80_, respectively.

Alves et al. [53] and Fagundes et al. [54] showed a reduction in enteric CH_4_ emissions that was of slightly greater magnitude than the one observed in our study. Namely, Alves et al. [53] achieved an average decrease in CH_4_ emissions of 32% by supplementing dairy cows with 120 g of *Acacia mearnsii* tannin extract. Likewise, Fagundes et al. [54] reported that diets containing 1.25% and 2.5% *Acacia mimosa* tannin extract reduced CH_4_ emissions in Nellore cattle by 17% and 33%, respectively, also showing the linear dose response described by Jayanegara et al. [9]. Two possible mechanisms underlying tannin-induced CH_4_ mitigation have been postulated, including direct inhibition of methanogenic micro-organisms (mainly Archaea and Protozoa), and consuming substrates for methanogenesis, primarily H_2_, to form stable complexes with proteins and carbohydrates, limiting their digestibility [8,50,52]. Some authors suggest causality between the mode of action and the form of tannins [9,10]. Therefore, the larger magnitude of enteric CH_4_ reduction recorded by both Alves et al. [53] and Fagundes et al. [54] compared to our study results could be explained by the different form of tannin molecules. In the present trial, we used hydrolysable tannins (HT; chestnut extract), while the aforementioned studies employed CT (*Acacia* spp.). Battelli et al. [10] demonstrated in vitro that tannins have the potential to reduce CH_4_ emissions by 1.44% to 8.61%, depending on its molecular form, with CT exhibiting greater efficacy than the HT. Interestingly, both CT and HT had no significant effect on the archaeal and protozoan population in the study performed by Battelli et al. [10], suggesting another mode of action in reducing CH_4_ emissions, probably decreasing organic matter digestibility.

Contrasting findings exist regarding tannin-microbiome interactions. Jayanegara et al. [9] observed declines in fibrolytic bacteria (*Fibrobacter succinogenes*, *Ruminococcus flavefaciens*) and anaerobic fungi, while Witzig et al. [19] reported reduced abundance of *Methanobrevibacter* spp. Contrary to the previously mentioned study, Jayanegara et al. [9] showed the highest effectiveness of CNT in CH_4_ reduction after comparing equal amounts of two HT and two CT extracts during an in vitro experiment. The disparity in efficacy may also reflect differences in supplementation duration. Alves et al. [53] and Fagundes et al. [54] observed significant CH_4_ reductions after 21 and 15 days, respectively, suggesting CT exerts effects rapidly. In contrast, HT may require longer intervention periods, as evidenced by Aboagye et al. [7], who detected no CH_4_ reduction in beef heifers after 14 days of 2% *Castanea sativa* HT supplementation. Our 21-day trial may thus represent an intermediate timeframe insufficient for maximal HT efficacy, particularly given finding of Duval et al. [55] that maximal CH_4_ suppression with mixed tannins (quebracho + chestnut) required three months in dairy cows. No significant difference in CH_4_ emissions was observed between CNT_40_ and CNT_80_ groups, possibly suggesting a dose-dependent decrease in emissions with a potential plateau or rebound at higher doses [13].

The results from this study are consistent with previous study that has correlated CH_4_ mitigation with protein precipitation capacity of chestnut tannins [9]. Namely, as a consequence of the significant decrease in enteric methane emissions observed with the CNT diets, it is reasonable to expect that CNT compared with CNT_0_ led to changes in the ruminal fermentation pattern, with a decrease in dietary protein degradation and an increase in the production and availability of the gluconeogenic precursor propionate, as well as higher hepatic glucose release. In line with this, the addition of chestnut tannin extract reduced blood urea nitrogen concentration after incorporating it into the diet at both 0.18% and 0.36% of dietary DM, reflecting the suppressive effect of chestnut tannins on rumen protein degradation that we previously observed [23].

Our study results corroborate numerous studies demonstrating improved protein utilization efficiency with low-to-moderate CNT supplementation [32,56,57]. In contrast, during mid-lactation, despite these metabolic changes, cows on the CNT diets had a blood glucose concentration similar to that of CNT_0_, which may be explained by improved insulin sensitivity at this stage of lactation and/or insulin-stimulated increase in tissue uptake of glucose of CNT cows. Analysis of metabolic hormones in our study showed for the first time that cows supplemented with chestnut tannins had significantly higher insulin and growth hormone (GH) levels than cows in the control group during mid lactation. Similar results were reported by Li et al. [58], who investigated the metabolic effects of mulberry leaf flavonoids, which also belong to the group of plant-derived polyphenols. They reported that mulberry leaf administration enhanced the concentration of serum metabolic hormones, including insulin and GH, in Murrah buffaloes during heat stress, and attributed the observed endocrine profile changes to structural similarities of flavonoids with steroid hormones. However, the elevated mid-lactation insulin and GH concentrations following chestnut tannin supplementation mirror findings by Zhao et al. [59] and support the established relationship between intestinal amino acid availability and endocrine regulation [60]. Furthermore, the higher growth hormone concentration in serum of cows when the CNT_80_ diet was fed likely led to increased mobilization of lipid reserves, resulting in the higher concentrations of circulating NEFA. Moreover, the higher serum BHBA concentration in cows fed the CNT_80_ diet, along with NEFA and growth hormone, indicate greater utilization of NEFA by the liver for the production of ketone bodies. Although the BHBA concentration arises mainly from metabolism of NEFA, the increase in response to CNT_80_ diet is the result of the latter, plus the BHBA resulting from butyric acid absorbed by the rumen, which would be expected to be higher when the CNT diet was fed, as recently observed by Cappucci et al. [61].

Additionally, we assessed the release of the enzymes LDH and AST from cells in order to indirectly monitor the degree of lipid peroxidation and liver function. The concentrations of all blood biomarkers measured to monitor metabolism and liver functionality were within the typical ranges reported for mid-lactation cows [62]. However, despite having a higher NEFA and BHBA concentrations, the lower AST and LDH activities in both CNT_40_ and CNT_80_ cows confirm that the CNT diets fed did not affect liver functionality and antioxidant status.

The results of the present in vivo study showed a significant effect of chestnut tannin extract in increasing milk production in comparison with the control diet. This is in line with previous in vivo research that has assessed the effects of a similar product based on the extract of chestnut tannins in mid-lactation Holstein and crossbred dairy cows [31,32]. Moreover, in our study, production responses to chestnut tannins in the diet were lower than in previous research when a similar amount of product was fed to dairy cows through a TMR [32], which is probably related to the differences in dietary amino acids supply and/or in the balance for absorbed amino acids from the diets. Dietary chestnut tannins can modulate milk production response through direct and indirect mechanisms. As previously reported, and also in our experiment, chestnut tannins had no effect on DMI and milk lactose, which are two of the most important determinants of milk yield [32,33]. Therefore, these direct factors are probably not involved in the differences in MY and FPCM between groups after chestnut tannins supplementation. However, the indirect route of action mediated through altered lactogenic hormones could promote milk production in response to chestnut tannins in mid-lactation cows. Thus, it is reasonable to suggest that an increase in growth hormone with the CNT_40_ and CNT_80_ diets was responsible for increased milk yield in the present study because growth hormone is extremely important in the regulation of milk secretion in dairy cows. In other words, these observed improvements can be attributed to the enhanced blood flow and supply of digestible amino acids, which is consistent with the observation of Roy et al. [63], who found that the mammary blood flow and supply of essential amino acids increased in lactating sheep fed a diet containing condensed tannins. In addition, the higher insulin concentrations in response to CNT diets may account, in part, for the increase in milk yield, as reported previously by other studies [64,65,66]. Namely, insulin increases GLUT 1 and GLUT 8 expression in bovine mammary explants, thus providing evidence of a functional link between circulating insulin and mammary glucose uptake [67,68]. Therefore, acting on hormonal status chestnut tannins could indirectly promote milk production in mid-lactation cows.

Another fact that emerges from the results of this study is the higher protein but lower fat content in milk, in response to a higher inclusion rate of chestnut tannin extract. Some lactation studies have shown positive changes in the content of the milk protein when chestnut tannin extract was added to the diet [31,32] but not others [56,69]. The beneficial effects on milk protein after incorporating CNT extract in the diet at 0.36% of dietary DM is in accordance with previous research in which a similar amount of tannin extracts was fed to dairy cows [48]. However, these authors reported no significant effect on milk protein content when increasing tannin levels in the diet and explained it with a decreased supply of digestible rumen undegradable protein or metabolizable protein from reduced microbial protein. Thus, we consider that an enhanced supply of amino acids to the mammary gland, combined with higher circulating insulin of the CNT_80_ cows, may increase milk protein synthesis, at least in part, through phosphorylation of mTOR [70]. Furthermore, protein content findings might have been mediated through an increase in energy supply as reflected in the increase in feed efficiency measures (MY/DMI and FPCM/DMI). In addition, the positive effect of chestnut tannins on the protein content in milk may be related to their microbial activity. There is an in vivo study demonstrating the stimulatory effect of tannic acid, which is usually obtained from the aqueous extract of sweet chestnut, on the relative abundance of the *Succinivibrionaceae* microbial family [71], which positively correlates with the content of milk protein in dairy cows [72].

On the other hand, the increased mobilization of lipid reserves resulted in higher blood concentrations of NEFA and BHBA in the CNT_80_ group was not in agreement with milk fat content findings, since did not contribute to the higher milk fat content in these cows. Our observation that cows fed a diet containing the highest CNT level showed the lowest milk fat (%) contrasts with the findings of Aguerre et al. [48] who detected no effect of tannin extract level on milk fat (%). Moreover, despite the higher catabolism of adipose tissue, lower milk fat content observed for CNT_80_ diet might suggest lower availability of NEFA and BHBA for mammary gland. The latter could be the channeling of NEFA and BHBA to the peripheral tissue by insulin. However, several other mechanisms may be responsible for the observed milk fat content findings in CNT_80_ cows.

First, a proposed anti-methanogenic mechanism of tannins, potentially relevant to our findings, involves inhibition of fibrolytic microbes in the rumen, which may reduce acetate production. As acetate is a key precursor for milk fat synthesis, Alves et al. [53] suggested that its reduction could be linked to elevated circulating NEFA levels. Although we did not assess ruminal fermentation directly, the increase in NEFA and BHBA observed in CNT_80_ cows supports this possibility.

Second, the antibacterial effects of chestnut tannins may also have contributed to reducing the milk fat content. For instance, Buccioni et al. [17] revealed that CNT supplementation reduces the relative rumen abundance of the microbial genus *Butyrivibrio*, which is positively correlated with the fat percentage in dairy cows [72].

Lastly, the direct inhibitory effects of tannins on milk fatty acid synthesis should also be considered [73]. The lower fat content in the milk of CNT_80_ cows, compared to those of CNT_0_ cows, could be attributed to a reduction in sterol response element-binding protein 1 (SREBP 1) signaling mechanism. Namely, this reduction can be caused by an enhancement of the effect of tannins on conjugated linoleic acids (CLA) production as well as inhibitory biohydrogeneration intermediates [11,74]. In the current experiment, it appears that an increase in energy supply from inhibited methanogenesis and/or enhanced feed efficiency was used for production purposes and/or milk protein synthesis rather than milk fat synthesis.

Due to their capacity to bind proteins in a range of ruminal pH and reduce their degradation by micro-organisms, tannins, irrespective of the source, can reduce ruminal NH_3_ production and increase nonammonia nitrogen flow to the duodenum [9], along with lowering urinary nitrogen excretion and increasing fecal nitrogen excretion [57]. The shift in the nitrogen excretion route from the urine to the feces and reduction in MUN content benefit both cows and the environment. The results of the present study showed a significant effect of chestnut tannins in decreasing MUN concentrations in comparison with the control diet. Namely, feeding CNT extract linearly reduced MUN levels by 6.3% and 8.7% in CNT_40_ and CNT_80_ cows, respectively. This effect was expected since a close correlation between BUN and MUN has been reported [75], further highlighting the protein-binding properties of chestnut tannins that resulted in lower ruminal protein degradation. Similar MUN declining response in the presence of tannins was also reported by Aguerre et al. [57], with linear decrease when tannin content in the diet was increased from 0 to 1.80% of DM. Moreover, after testing the effects of different tannin sources (both HT and CT) on performance parameters in dairy cows, Zhang et al. [30] showed a clear reduction in MUN levels after both HT and CT consumption. In their review paper, Herremans et al. [28] stated a reduction in ruminal NH_3_-N and MUN by 16% and 8%, respectively, when cows were fed with tannins. However, the significantly reduced MUN and increased milk protein levels in response to CNT diets provide an opportunity for designing dietary strategies that might lead to an improvement in dairy herd protein nutrition as well as a reduction in N environmental pollution.

Other health benefits in response to the CNT extract used in this study could also be noticed in the lower SCC in milk, suggesting improved milk quality in these cows. For instance, CNT-supplemented cows compared with CNT_0_ had lower milk SCC, a result that highlights anti-inflammatory and antioxidant effects of chestnut tannins [34,69]. A previous study from our research group [23] has shown that feeding chestnut tannin extract increased IgG colostrum levels. Therefore, improved milk quality could also be explained by the positive effects of chestnut tannins on cows’ immune function and/or a lower bacterial contamination of milk in these cows.

## 5. Conclusions

The use of chestnut tannin extract in dairy cows’ diet can address several aspects of production. Supplementing chestnut tannins to lactating Holstein dairy cows during mid-lactation is effective not only in lowering the enteric CH_4_ emissions, but also in improving the lactation performance, largely dependent on the dosage used in our study. Hormone analyses in blood suggest that the beneficial effect of feeding this tannin extract on the milk production of mid-lactation cows is due, in part, to favorable alterations of the hormonal status. Furthermore, although adding 0.36% chestnut tannins to the diet reduced milk fat, it had a positive effect on milk protein content. The results obtained in this study indicate a promising potential of plant extracts, such as chestnut tannin extract, in gaining a greater role in the dairy feed additive industry. Future research is needed to investigate the long-term effects of chestnut tannins on milk yield and persistency during mid-lactation.

## Figures and Tables

**Table 1:** Ingredients and chemical composition of the diet for cows in mid-lactation.

Diet	Midlactation
Ingredient (g/kg of DM)	
Corn silage	411.87
Alfalfa silage	110.89
Alfalfa hay	20.37
Molasses	33.95
Corn grain	139.40
Barley	59.74
Soybean meal (44% CP)	97.76
Sunflower meal (35% CP)	92.60
Sodium chloride	3.15
Sodium bicarbonate	5.40
Calcium carbonate	9.01
Vitamin Mineral Mix	4.88
Protected fat (99% palmitic acid)	10.98
DMI (kg/day)	22.09
Chemical composition	
NEL (Mcal/kg of DM)	1.66
CP (g/kg ofDM)	16.67
RDP (g/kg ofCP)	10.60
RUP (g/kg of CP)	6.07
MP (g/kg of DM)	106.27
aNDF (g/kg of DM)	304.79
ADF (g/kg of DM)	197.56
NFC (g/kg of DM)	408.31
Ether Extract (g/kg of DM)	39.00
Ca (g/kg of DM)	89.94
P (g/kg of DM)	41.96

DM—Dry matter. CP—Crude protein. DMI—Dry matter intake. NEL—Net energy for lactation. RDP—Rumen degradable protein. RUP—Rumen undegradable protein. MP—Metabolizable protein. aNDF—Amylase-treated neutral detergent fiber. ADF—Acid detergent fiber. NFC—Non-fiber carbohydrate.

**Table 2:** The effect of chestnut tannins supplementation on indicators of CH_4_ emissions in mid-lactation dairy cows.

Parameter (Unit)	Treatments			SE	ANOVA *p*-Value	Contrasts (*p*-Value)	
	CNT_0_	CNT_40_	CNT_80_			L	Q
CH_4_ production (ppm)	93.4 ^a^	72.1 ^b^	74.2 ^b^	3.6	<0.001	0.001	0.011
CH_4_ yield (ppm/kg DMI)	4.35 ^a^	3.31 ^b^	3.42 ^b^	0.16	<0.001	<0.001	0.008
CH_4_ intensity (ppm/kg FPCM)	4.56 ^a^	3.24 ^b^	3.37 ^b^	0.20	<0.001	<0.001	0.005

CNT_0_—No supplemented cows. CNT_40_—Cows supplemented with 40 g of chestnut tannin extract (1.8 g/kg of DM). CNT_80_—Cows supplemented with 80 g of chestnut tannin extract (3.6 g/kg of DM). ^a, b^ Means with different letters between columns differ significantly (*p* < 0.05). SE—Standard error. L—Linear contrast. Q—Quadratic contrast.

**Table 3:** The effect of chestnut tannins supplementation on blood biomarkers of metabolism in mid-lactation dairy cows.

Parameter (Unit)	Treatments			SE	ANOVA *p*-Value	Contrasts (*p*-Value)	
	CNT_0_	CNT_40_	CNT_80_			L	Q
Glucose (mmol/L)	2.97 ^a^	2.89 ^a^	3.01 ^a^	0.10	0.711	0.862	0.444
BUN (mmol/L)	4.56 ^a^	3.94 ^b^	3.74 ^b^	0.18	0.006	0.005	0.176
NEFA (mmol/L)	0.28 ^a^	0.32 ^a^	0.39 ^b^	0.02	0.010	0.002	0.504
BHBA (mmol/L)	0.83 ^a^	0.87 ^a^	1.01 ^b^	0.04	0.009	0.004	0.295
Insulin (IU/mL)	11.5 ^a^	13.7 ^b^	16.1 ^c^	0.49	<0.001	<0.001	0.977
Growth hormone (ng/mL)	1.17 ^a^	1.47 ^b^	1.56 ^b^	0.13	0.085	0.027	0.537

CNT_0_—No supplemented cows. CNT_40_—Cows supplemented with 40 g of chestnut tannin extract (1.8 g/kg of DM). CNT_80_—Cows supplemented with 80 g of chestnut tannin extract (3.6 g/kg of DM); BUN—Blood urea nitrogen. BHBA—Beta-hydroxybutyric acid. NEFA—Non-esterified fatty acids. ^a–c^ Means with different letters between columns differ significantly (*p* < 0.05). SE—standard error. L—Linear contrast. Q—Quadratic contrast.

**Table 4:** The effect of chestnut tannins supplementation on blood biomarkers of liver function in mid-lactation dairy cows.

Parameter (Unit)	Treatments			SE	ANOVA *p*-Value	Contrasts (*p*-Value)	
	CNT_0_	CNT_40_	CNT_80_			L	Q
Albumin (g/L)	34.7 ^a^	33.6 ^a^	36.5 ^a^	1.52	0.388	0.223	0.281
Total bilirubin (µmol/L)	1.61 ^a^	1.59 ^a^	1.68 ^a^	0.10	0.761	0.408	0.747
AST (U/L)	108.7 ^a^	88.5 ^b^	92.0 ^b^	5.49	0.037	0.043	0.090
LDH (U/L)	2126.2 ^a^	1728.6 ^b^	1707.5 ^b^	104.7	0.011	0.007	0.141

CNT_0_—No supplemented cows. CNT_40_—Cows supplemented with 40 g of chestnut tannin extract (1.8 g/kg of DM). CNT_80_—Cows supplemented with 80 g of chestnut tannin extract (3.6 g/kg of DM); AST—aspartate aminotransferase. LDH—Lactate dehydrogenase. ^a, b^ Means with different letters between columns differ significantly (*p* < 0.05). SE—Standard error. L—Linear contrast. Q—Quadratic contrast.

**Table 5:** The effect of chestnut tannins supplementation on milk production traits in mid-lactation dairy cows.

Parameter (Unit)	Treatments			SE	ANOVA *p*-Value	Contrasts (*p*-Value)	
	CNT_0_	CNT_40_	CNT_80_			L	Q
Milk yield (kg/day)	26.2 ^a^	29.0 ^b^	29.3 ^b^	0.63	0.003	<0.001	0.113
FPCM (kg/day)	20.6 ^a^	22.8 ^b^	22.8 ^b^	0.49	0.003	0.003	0.115
FE_MY_ (milk kg/kg of DMI)	1.23 ^a^	1.33 ^b^	1.34 ^b^	0.03	0.014	0.003	0.159
FE_FPCM_ (kg of FPCM/kg of DMI)	0.96 ^a^	1.05 ^b^	1.05 ^b^	0.03	0.015	0.013	0.171
Milk fat (g/100 g)	4.21 ^a^	4.13 ^ab^	4.03 ^b^	0.41	0.023	<0.001	0.919
Milk protein (g/100 g)	3.45 ^a^	3.50 ^ab^	3.54 ^b^	0.02	0.001	0.004	0.692
Milk lactose (g/100 g)	4.64 ^a^	4.66 ^a^	4.70 ^a^	0.01	0.094	0.966	0.733
MUN (mg/dL)	12.7 ^a^	11.9 ^b^	11.6 ^b^	0.11	<0.001	0.130	0.768
SCC (Log_10_ SC/mL)	2.50 ^a^	2.31 ^b^	2.17 ^b^	0.05	<0.001	0.001	0.964

CNT_0_—No supplemented cows. CNT_40_—Cows supplemented with 40 g of chestnut tannin extract (1.8 g/kg of DM). CNT_80_—Cows supplemented with 80 g of chestnut tannin extract (3.6 g/kg of DM). FPCM—Fat- and protein-corrected milk. FE_MY_—Milk yield feed efficiency. FE_FPCM_—FPCM feed efficiency. MUN—Milk urea nitrogen. ^a, b^ Means with different letters between columns differ significantly (*p* < 0.05). SE—Standard error. L—Linear contrast. Q—Quadratic contrast.

## Data Availability

The data presented in this study are available upon request from the corresponding author and are available at https://mitimetcattle.vet.bg.ac.rs/ (accessed on 18 June 2025).
